# It's all about recognition! Qualitative study of the value of interpersonal continuity in general practice

**DOI:** 10.1186/1471-2296-10-47

**Published:** 2009-06-26

**Authors:** Heidi Bøgelund Frederiksen, Jakob Kragstrup, Gitte Dehlholm-Lambertsen

**Affiliations:** 1 Research Unit of General Practice, Institute of Public Health, University of Southern Denmark, JB Winsløws Vej 9A, 5000 Odense C, Denmark; 2 Odense University Hospital, Sønder Boulevard 29, 5000 Odense C, Denmark

## Abstract

**Background:**

Continuity of care has traditionally been regarded as a core quality of general practice, but the long-term doctor-patient relationship has been put under pressure. In many places practices are expanding, with larger teams and more registered patients, thereby threatening the possibility of patients staying with their own general practitioner (GP). GPs often take it for granted that interpersonal continuity is valuable. However, little is known about how patient satisfaction is related to interpersonal continuity. The purpose of this study is to explore the creation of patient satisfaction or dissatisfaction in the interpersonal relation with the GP, and in a comprehensive way to investigate how this is related to continuity of care.

**Methods:**

Qualitative study based on 22 interviews with patients from two practices in Denmark. A total of 12 patients saw a regular doctor and 10 saw an unfamiliar doctor. The patients were selected after an observed consultation and sampled purposefully according to reason for encounter, age and sex. Interpretative phenomenological analysis (IPA) was used to study how patients perceive meeting either a regular or an unfamiliar GP. The analysis explored the patients' perception of their interpersonal relationship with their GP, and interpreted the accounts by using social psychological theories.

**Results:**

A long-term continuous relationship with the GP could be satisfactory, but it could also be the reverse. The same pattern was shown in case of an unfamiliar GP. Therefore, patient satisfaction and interpersonal continuity were not causally related. On the contrary, there was a general pattern of how the satisfactory and trustful doctor-patient relationship from the patients' point of view could be created, maintained or destroyed. A pattern where the process of recognition, by respecting and remembering, on the one hand created and maintained satisfaction while humiliation on the other hand destroyed satisfaction in the relationship.

**Conclusion:**

It was not valuable to have a continuous relationship unless the GP recognized the patient. The social psychological concept of recognition had two different meanings and the GP had to do both, respect and remember the patient, in order to create and sustain the trustful relationship. The added value of interpersonal continuity had to be combined with recognition.

## Background

Continuity of care has traditionally been regarded as a core quality of general practice [1] and it appears to be important to a majority of patients [2-5]. Continuity of care has therefore been divided into three different aspects: Informational, management and relational continuity or in other words interpersonal continuity [6]. Patients value different aspects of continuity for different reasons [5,7-9] and patients suffering from chronic diseases often give high priority to interpersonal continuity [3,10,11]. Great efforts have been made to clarify the importance of interpersonal continuity but a theoretical evidence base for interpersonal continuity is unfortunately lacking. It is still not clear how interpersonal continuity makes a difference to the quality of care in general practice. Surveys find a positive relationship between interpersonal continuity of care and patient satisfaction, but it is difficult to tell whether continuity leads to satisfaction or satisfaction leads to continuity [4,12]. It is argued that general practice needs to turn to the sciences of human behaviour to develop a theoretical basis for the relationship between interpersonal continuity and patient satisfaction [13,14]. The aim of the article is to explore the creation of patient satisfaction or dissatisfaction in the interpersonal relation with the GP and in a comprehensive way investigate how this is related to continuity of care. Longitudinal or provider continuity implies a pattern of visits [15]. We have used the term interpersonal continuity for the relational aspects of this. It does not directly address positive or negative aspects of the relationship between the patient and the provider.

## Methods

### Setting and recruitment

The study was carried out in two general practices in Denmark. Six GPs participated: Three GPs were regular GPs with several years of experience in the same practice, and three were trainees, who worked 6 months in a practice. The aim of the study was to investigate the relationship between interpersonal continuity and satisfaction. We were therefore questioning the value of interpersonal continuity instead of taking it for granted, and we assumed we would see similarities and differences more clearly by comparing patients' experiences with regular and unfamiliar GPs. A regular GP is defined as the GP the patient usually sees and an unfamiliar GP is defined as a GP seen for the first time. The researcher observed consultations on a sample day. A total of 22 patients were recruited for interviews, 12 patients who were seeing their regular GP and 10 patients who were seeing the GP for the first time. The patients were selected after the consultation and sampled purposefully [16] according to different reasons for encounter, age, sex and familiarity with the GP (Table 1).

**Table 1:** Characteristics of interviewees

	n = 22 *	**Practice 1**	**Practice 2**
**Sex**			
Male	10	4	6
Female	12	6	6
			
**Age**			
18–35	6	2	4
36–54	8	3	5
55–82	8	4	4
			
Consulted GP **			
Regular	12	5	7
Unfamiliar	10	5	5
			
**Reason for encounter**			
Acute	11	6	5
Non-acute	11	4	7

* Numbers out of 22. ** An unfamiliar GP is a GP seen for the first time

### Interviews

A total of 22 patients were interviewed. Two patients from the first practice were interviewed twice in order to get a thorough understanding of the patients' accounts. The selected patients were phoned a few days after the consultation and asked if they wanted to participate. Interviewees were told that information provided by them would not be reported to their GP. Three men, aged 18–35 years, declined to participate. The interviews took place in the patients' home. The patients were asked to assess the observed consultation. They were then asked to compare the consultation with their experience with their regular GP and other GPs (Table 2). In order to maintain anonymity all GPs appear in the article as men. We compared consultations with regular as well as unfamiliar GPs, and from this, we derived the components that generated satisfaction at the first meeting and over time.

**Table 2:** The semi-structured interview guide covered the following themes and questions

**1. Personal information about the patient**	• Tell me about yourself – age, family, job and illness(es).
	• For how long have you been a patient of this GP?
	• Have you been a patient of other GPs?
**2. Description and assessment of the observed consultation**	• Describe the consultation with the GP, where I was present.
	• What is your assessment of the encounter? Try to find words to describe it.
	• Were you satisfied with the GP?
	• What does it take for you to be satisfied with your GP?
**3. Experience with this GP and GPs in general**	• Do you know this GP?
	• If no: Would you see the same GP again?
	• Do you have a regular GP?
	• How often do you visit your healthcare center?
	• Who do you consult? Your regular GP or an unfamiliar GP?
	• Describe some good experiences at the GP.
	• Describe some bad experiences at the GP.
**4. The importance of relational continuity**	• Is it important to you that your GP knows you?
	• If yes, explain how and when it is important.
**5. Comparison between satisfaction with the GP and the health system in general**	• Do you have any experiences with other areas of the healthcare system?

### Analysis

A phenomenological approach called interpretative phenomenological analysis (IPA) [17] was considered appropriate in order to study how patients perceive meeting either a regular or an unfamiliar GP. The interviews were fully transcribed. IPA differs from descriptive phenomenology with more emphasis on interpretation, and thematic analysis is the principal analytical approach [[17]:110]. The analysis begins with a single case and proceeds through the following stages. At stage one, the analysis is concerned with making sense of the participants' world, and therefore works through the transcripts several times. At stage two, emerging themes are noted. At stage three, themes are listed and the analyst attempts to identify common links between themes and to reorder them in a more analytical or theoretical way. At the final stage four, themes are appropriately named and each theme is linked to the originating text through reference to specific quotes. Once these stages have been completed for one interview, themes generated from the analysis of the first interview guide the analysis of the next interview. This approach emphasises close examination of negative or contrary instances while aiming for conclusions that can be generalised to the study population. The meaning of themes will be linked to appropriate social psychological literature in order to interpret the theoretical meaning of the subjective accounts.

### Ethics committee

The Regional Scientific Ethics Committee was informed of the study, but they did not find that it came under the provisions of Danish act on biomedical studies and approval by the Committee was therefore not required.

## Results

The analysis showed two core, conflicting themes that either generated patient satisfaction or patient dissatisfaction. The themes were all about either being taken seriously or not taken seriously. In the beginning of the analysis the themes were more descriptive than theoretical, but by linking to social psychological theories [18] it became possible to develop a theoretical pattern for the relationship between interpersonal continuity and patient satisfaction. By means of the two social psychological concepts, *recognition *and *humiliation*, the analysis of the pattern of creation and destruction of satisfaction became clear. Recognition is a relationship concept – an attitude expressed through interaction [18,19]. On the one hand, the process of recognition, by respecting and remembering, created and maintained satisfaction while humiliation on the other hand destroyed satisfaction in the relationship. Humiliation is in its everyday sense a strong word and it has to be understood in a theoretical frame of reference, i.e. as the opposite of recognition [20].

### Recognition

All the interviewed patients expressed satisfaction with the interpersonal relationship with the GP in the observed consultation. According to the interviewed patients the crucial points for satisfaction with the relationship were the GP's ability to talk seriously about their problem as well as the fact that the GP 'saw' them;

*"He listened to what I was saying, and he took me seriously. So I felt that it was I who was important" (Marianne, 29 years old, first encounter)*.

'To be taken seriously' is a complex process with constituent parts all forming part of a whole. With a comprehensive term, the process of taking a patient seriously is defined as recognising the patient and his or her application. The term recognising has two different meanings, respecting and remembering, and in this context it means being respected by the GP. The patient experienced that she was taken seriously, if the GP was able to recognise the patient by listening, understanding, confirming and accepting. It satisfied the patient and trust was established;

*"I wanted the GP to take it seriously. It is not a serious problem; it is nothing that I will die of or be injured from in any way. It is purely cosmetic, right? But the fact that he understood that it was not something that he should avoid doing something about. It should not just be brushed aside. I realise that it is not important, but it is still important to me. Therefore, I think that it was a good consultation. It was satisfactory" (Ninna, 26 years old, first encounter)*.

The above patient was seen by a GP she did not know in advance. It was the GP's ability to recognise (respect) her combined with his professional ability that created the immediate satisfaction.

The observations in the consultation confirmed the patients' statements that when the GP, in a non-verbal way, indicated acceptance and understanding by nodding and keeping eye contact, the patient felt recognised (respected). If the GP focused on the patient by for instance turning his body directly towards the patient and not towards the computer, his body language signalled presence. It made the patient feel important;

"I felt that I was in good hands. I had the feeling that I should sit here, and for the next ten minutes, I would be the subject of importance, and indeed, not all GPs are capable of sending that signal. He did this through his behaviour. He signalled that he was interested in what I was saying, and he was calm". (Lene, 56 years old, first encounter)

One patient described his first meeting with the GP this way;

"My impression of him was that he was very obliging. He welcomes you in a pleasant way and says hello and is kind and obliging. So you immediately feel welcome, and I think that he was nice to talk to. He keeps eye contact and is attentive. So my impression is that he is nice to talk to, he seems trustworthy". (Frederik, 39 years old, first encounter)

Satisfaction and trust were constructed contextually in a dynamic process between the GP and the patient, and it did not depend on longitudinal continuity. On the contrary, it depended on the fact that the GP took the patient seriously at the actual encounter.

### Humiliation

All of the 22 interviewees could remember an unsatisfactory encounter with a GP. The patients told that they felt humiliated if the GP did not take them seriously. This would happen if the GP ignored, insulted or ridiculed the patient. Therefore all of the negative experiences dealt with the opposite of recognition, i.e. humiliation. The study showed that the first encounter between the GP and the patient was crucial to many of the patients. If the patient felt humiliated, he or she did not want to consult the same GP again;

*"I went to see the GP about my knees. The GP's conclusion was that I had to find something else to do, and honestly, I thought that it was a strange thing to say, because if you are told that you cannot work anymore, then what should I do? He cannot just say that I should find something else to do because you can't do that at my age. No, I don't want to consult him again. I do not trust him at all, so I would not like to consult him again*".

Interviewer: So the trust is gone?

"Yes, honestly, I think it is. It would be difficult for me to believe what he was saying the next time I consulted him. It would. (Erik, 32 years old, first encounter).

If the GP humiliated the patient by not taking him or his problem seriously, the patient lost confidence in the GP. The overall pattern of recognition versus humiliation was most obvious at the first encounter. It may create the basis for a satisfactory, continuous, trustful relationship, but if the encounter generated distrust, it created no basis to build on.

### Association between interpersonal continuity and satisfaction with the relationship

There were ten patients who saw a trainee and all of them were satisfied with the relationship in the consultation. Two of the ten patients did not care who they were to see next time. They were both under the age of 30, and nothing "serious" was wrong with them. For the other eight patients interpersonal continuity was important. Two of the other eight patients would like to continue seeing the trainee because he had started the course and they wanted him to follow up. The other six patients said that they would like to see the trainee again, if they had a minor problem. But they preferred to see their regular GP, if they had a severe problem.

When a good relationship was created with the regular GP, it was valuable for the patients to maintain it. A total of 12 out of 22 interviewees saw their regular GP. They all had a good and trustful relationship with him and preferred to maintain it. They had several reasons for that. It strengthened the feeling of being taken seriously, if the GP remembered the patient;

I think that the better you know your GP, the better you sense that he is taking you seriously. This may be because the GP you consult on a regular basis will of course be better at remembering your situation. The very fact that the GP is able to continue to talk about your disease immediately makes you automatically feel that he is taking you seriously, because he remembers what we talked about the last time I consulted him. (Dennis, 48 years old, 9 years with the same GP)

Another valuable aspect of the continuous relationship was that it provided the patients with a feeling of security. In particular, the patients who were in long-term courses of a disease or who suffered from a chronic disease most clearly expressed their need for security;

*I would feel unsecure not consulting a regular GP. I would feel unsecure if one was to continuously meet new faces and inform them. Even though they have our records, you will never have the same contact; and thoroughness, if they have not followed you for many years... and he performs the same examinations every time; and then, it also makes me secure that he knows, and that I know, what is going to happen to me." (Anna, 58 years old, patient with a weak back, 31 years with same GP)*.

According to the interviewees, it also generated improved coherence in the treatment, and it was a relief not to be forced to tell the medical history over and over again. It made it of special value to sustain the relationship;

I always make an appointment with Peter. I like the continuity. Then I don't have to start with Adam and Eve every time. (Lene, 56 years old, chronically ill, 5 years with same GP)

Many statements described that it was especially satisfying when the patient felt that the GP was interested in the patient as a person, which was possible if the GP knew the patient;

"I seldom associate it with a positive thing to see the GP, but it is a little more comfortable to go there when, at least, you have felt that he is interested in you. I think it is nice when he remembers and recognises you when you go there. This makes you a little more relaxed". (Erik, 32 years old, 32 years with the same GP)

It was therefore very satisfying to be remembered by the GP;

*When we consult either Antonsen or Larsen, we are recognised, they know who we are; they may very well skim the records on the computer screen before we enter, but they always ask how things went with this and that. This makes me feel secure. It seems like, well, we are in good hands here, they remember you, they know what it is all about, and that's the way it should be. (Bente, 57 years old, 3 years with same GP)*.

The GP should respect and remember the patient; as a person as well as in relation to illness. The term recognise has two different meanings, *respect *and *remember*, and if the GP did both, interpersonal continuity was especially valuable to the patients. It created a good and trustful relationship to the GP.

### Association between interpersonal continuity and dissatisfaction with the relationship

However, the repeated visits made the GP-patient relationship vulnerable. If interpersonal continuity had to be valuable, the meaning of recognition had to include remembering. The patients expected to be remembered as a person, and they became very disappointed if the GP did not remember them;

When we consulted Hansen, it was like we were there for the first time; he hardly knew who you were. I didn't feel that he took me seriously. He did not respect me, and then he talked about his own problems. It is a relationship of trust; it's a question of respect. We need to be secure. (Bente, 57 years old, 20 years with her former GP)

Remembering was a decisive element in being able to maintain satisfaction by interpersonal continuity. If the GP did not remember the patient, then interpersonal continuity lost its value;

*I don't think that Sørensen ever got to know me. Well, I was kind of alienated when he saw me. When I was called in from the waiting room, it was almost like he saw me for the first time. I had been attached to this health care centre for six years, so he should know who I am, shouldn't he? I think that he (the new GP) listens more to what I say and is very focused on the fact that we should try to find out what is wrong. Whereas I sensed that Sørensen would not have done that at all". (Katrine, 72 years old, 7 years in same healthcare centre)*.

Even though the above patient had a continuous relationship with her former GP, she was dissatisfied. She felt objectified. Therefore, she changed after 6 years to one of the other GPs in the same health centre whom she had seen by chance. The new GP 'outmatched' the continuous relationship because he remembered her, "*listened more"*, and *focused on *taking her inquiry seriously as opposed to the former GP.

All of the negative experiences dealt with the opposite of recognition (remembering and respecting), i.e. humiliation. Humiliation was an overall term for the negative experiences. There were numerous examples of the relationship never becoming satisfactory if the patient felt objectified, insulted, ignored or ridiculed even though the patient continued with the same GP;

*"I had actually suffered from a bad leg for years, but I damn well did not mention it to Hansen anymore. He had laughed at me once, and he should not be allowed to do that again, should he? There have been a lot of such examples; that he almost laughed and started talking about the birds in the garden, how many different kinds he had and that he could hardly manage to take care of the garden." (Bente, 57 years old, 20 years with her former GP)*.

The above patient felt exposed to ridicule. She had seen this GP for 20 years, even though she was dissatisfied. This applied to some of the other patients. They told about long-term relationships where they had been dissatisfied without changing GPs. However, the patient could feel so offended in a specific consultation that there was no other solution than to change;

*"I changed because he said that I was hysterical. I had a problem of sweating a lot and he examined me, but ended up telling me that I was hysterical. There are certain things you do not want to hear when you go to the GP. It is something you want him to take seriously". (Pernille, 29 years old, 8 years with former GP)*.

For a long time, the above patient had been dissatisfied. But it was not until she felt that the GP offended her specifically that she changed. Another patient felt insulted in a specific consultation because the GP did not take his suggestion seriously, and afterwards he changed GP;

*"I went to my former GP, and asked if it could be this disease, my mother had. He said; no, it is not, I guarantee. Then I asked for some tests and he just said; if you insist! But I was right. Then the trust was gone." (Søren, 72 years old, 15 years with former GP)*.

Even though the GP-patient relationship was characterised by dissatisfaction, the relationship could still be a long-lasting one.

## Discussion

### Summary of main findings

When the interviewees were asked what they would prefer in theory: a regular GP or a new GP from consultation to consultation, the majority preferred a regular GP. However, the results showed that all the patients who had consulted an unfamiliar GP were satisfied with the consultation and that several of the patients who had a regular GP were dissatisfied. By comparing the patient's ideal statement with the actual experience in the consultations, we could thus conclude that interpersonal continuity is not a value in itself. A long-term continuous relationship could be satisfactory, and the meeting with an unfamiliar GP could be unsatisfactory. But the study also showed that the patients could have a long-term continuous relationship with the GP without being satisfied, and that the meeting with an unfamiliar GP could be satisfactory. Patient satisfaction and interpersonal continuity were thus not always related. Instead the analysis showed a pattern where recognition, by both respecting and remembering, on the one hand created and maintained satisfaction while humiliation on the other hand destroyed satisfaction. The combination of recognition (respect and remembering) with interpersonal continuity generated an added value in the interpersonal doctor-patient relationship that could not be generated at the first encounter.

### Strengths and limitations of the study

The study provides new knowledge on how interpersonal continuity is able to improve patient satisfaction. So far, research has not yet clarified the exact correlation between interpersonal continuity and patient satisfaction [13]. Studies have reported associations between the two concepts [4,12], but no studies have shown how satisfaction is created in one off versus repeated consultations. According to the present study patients could also be satisfied with an unfamiliar GP and another research even shows that certain patients prefer discontinuity [21]. The strength of this study was therefore, that it did not take for granted that interpersonal continuity alone is positive to patients. This opened up the possibility of varying the previous understanding of interpersonal continuity and reporting a pattern of creation and destruction of satisfaction.

The weakness of the study is that we did not observe any consultations with humiliation. The analysis of humiliation is based solely on the patients' descriptions of dissatisfying consultations. However, the patients compared good and bad consultations, and from their descriptions we saw how dissatisfaction was generated by ignoring or insulting i.e. humiliation in the consultations. There may be a difference between patients younger than 30 and older than 30 according to the preference of a continuous relationship. The two patients who did not care about having a regular GP were both under 30 years. This could indicate that the possibility of creating a continuous relationship is less important to younger patients. This does not, however, change the fact that the general theme creating patient satisfaction in the relationship for all of the 22 patients was to be taken seriously in the actual consultation, in other words to be recognised. Another limitation of the study is the small number of participants. It is a qualitative study and there may possibly be bias in the selection of the patients.

### Comparison with existing literature

The study was questioning the value of interpersonal continuity instead of taking it for granted that it was positive. Therefore this study avoided the term relational continuity [6], now improved to relationship continuity because it has been defined as building on accumulated knowledge of patient preferences and interpersonal trust based on experience of past and positive expectations of future care [22]. We also wanted to investigate the possible non-positive connotations of the relationship. The results are consistent with several other studies showing a connection between interpersonal continuity and patient satisfaction [3,4]. One study showed that trust was more important than interpersonal continuity according to levels of satisfaction but did not explain why [23]. Another study showed that *"finding the right GP to talk to may be more important for patients than sticking with the same GP" *[24]. The new finding of this study is that it illuminates the relationship between interpersonal continuity, trust and patient satisfaction. This study shows that longitudinal continuity does not in itself lead to satisfaction. Continuity of care does matter [22] but only combined with recognition. Then it becomes relationship continuity [22]. The process of creating a relationship with a GP is an active, dynamic process [25] and the patients want "to be taken seriously". To be taken seriously created trust, which other studies have also shown [26,27], but they did not explain why, in theoretical terms. There are no studies, either, that show what it means to the value of interpersonal continuity "to be taken seriously". The main result in this study was that it was not valuable to have a continuous relationship unless the GP was recognising in his behaviour, i.e. was listening, understanding, confirming and accepting the patient. The process of recognition contains both empathy [28] and good communication skills [29], but it is more than that. It is a fundamental respect for the experience perspective of the opposite party [30]. This was necessary both in order to generate a good contact with the patient in the first encounter, and in order to sustain the relationship. The need for recognition, by both respecting and remembering, was reflected in the fact that the relationship was described as unsatisfactory if the GP did not remember the patient. According to the sociologist, Axel Honneth [18], recognising as a concept is crucial for generating identity today. The responsibility for self-realisation to a great extent rests on the individual because in today's cult of the individual we have lost traditional hierarchies and overt social divisions. The human being is created, according to Honneth, relationally, and recognition (respecting and remembering) is a crucial component in the creation of a good interpersonal relation. It is a complex process to be acknowledging towards the patient, and it requires the GP to be constantly emotionally available. Therefore, the good doctor-patient relationship requires reflection by the GP which another study has shown [19], but this study shows that recognition applies to all kinds of patients, not only patients suffering from chronic diseases. The unsatisfactory doctor-patient relationship is, according to the patients, a relationship characterised by ignorance, insult and lack of listening from the GP's part, i.e. humiliation. This is also seen in other empirical studies [31,32]. Ignorance was by the patient considered to be a humiliation of the patient's identity. The results are in agreement with an article describing humiliation in theoretical terms [20]. According to that article, the GP may, inadvertently, humiliate the patient, because the medical profession legitimises objectification in the doctor-patient relationship. Our study confirmed that. If the GP humiliates the patient through indifferent behaviour, the relationship with the GP is not satisfying even though the relationship continues.

### Implications for future research and clinical practice

The study showed that GPs cannot take for it granted that their patients are satisfied, even though there is a long-term relationship between them. Some patients stayed even though they were dissatisfied. Future research is needed to explore why patients stay with a GP they feel humiliated by. A recently published study confirms that good communication skills of GPs, whether they are familiar with the patient or not, enable patients to discuss any issue [33]. However, future research is needed to explain why patients who were satisfied with the trainee often preferred to see their regular GP. This study showed that a regular GP is particularly valuable to the patients as long as the GP is recognising in his behaviour. This is vital knowledge to GPs as well as to administrators. The tendency is to expand the sizes of practices without considering how to maintain interpersonal continuity. This would be a reduction of quality, which has also recently been shown [34].

## Conclusion

If the patients felt recognised in the consultation they were satisfied with the relationship, also if it was with an unfamiliar GP. However, if the patients were satisfied with their regular GP, they often preferred to see this GP continuously, and interpersonal continuity became valuable. It created a sense of security. But if the patients felt humiliated by their GP, or if the GP did not remember them as a person, interpersonal continuity had no added value. The theoretical concept of recognition has two different meanings and the point is that the GP has to do both, respect and remember the patient, in order to create and sustain a satisfying relationship.

## Competing interests

The authors declare that they have no competing interests.

## Authors' contributions

HBF carried out the design of the study, collected the data, performed the data analysis and drafted the manuscript. JK and GDL participated in the design of the study and the data analysis. All authors read and approved the final manuscript.

## Pre-publication history

The pre-publication history for this paper can be accessed here:


